# Crystal structure of tetra­kis­(tetra­hydro­furan-κ*O*)bis­(tri­fluoro­methane­sulfonato-κ*O*)iron(II)

**DOI:** 10.1107/S2056989019013094

**Published:** 2019-09-27

**Authors:** Charl F. Riemersma, Emily C. Monkcom, Robertus J. M. Klein Gebbink, Martin Lutz

**Affiliations:** a Organic Chemistry and Catalysis, Debye Institute for Nanomaterials Science, Faculty of Science, Utrecht University, Universiteitsweg 99, 3584 CG Utrecht, The Netherlands; b Bijvoet Center for Biomolecular Research, Crystal and Structural Chemistry, Faculty of Science, Utrecht University, Padualaan 8, 3584 CH Utrecht, The Netherlands

**Keywords:** crystal structure, high-spin iron(II), split-mosaic model

## Abstract

The title compound, [Fe(CF_3_SO_3_)_2_(C_4_H_8_O)_4_], is octa­hedral with two tri­fluoro­methane­sulfonate ligands in *trans* positions and four tetra­hydro­furane mol­ecules in the equatorial plane. By the conformation of the ligands the complex is chiral in the crystal packing. The compound crystallizes in the Sohncke space group *P*2_1_2_1_2_1_ and is enanti­omerically pure. The packing of the mol­ecules is determined by weak C—H⋯O hydrogen bonds. The crystal studied was refined as a two-component inversion twin.

## Abstract 2

The title high-spin iron(II) complex is six-coordinated with two tri­fluoro­methane­sulfonato and four tetra­hydro­furan ligands. It is isostructural with the corresponding Co, Ni and Zn complexes known from the literature.

## Chemical context   

The tri­fluoro­methane­sulfonato anion is usually weakly coordinating to metals, and the salts thereof are consequently important starting compounds for the exchange with other ligands. In an attempt of such a synthesis on iron(II) we obtained the starting material back with tetra­hydro­furan (THF) mol­ecules from the solvent completing the sixfold coordination environment. The overall composition of the title compound (I) is then [Fe(CF_3_SO_3_)_2_(C_4_H_8_O)_4_].
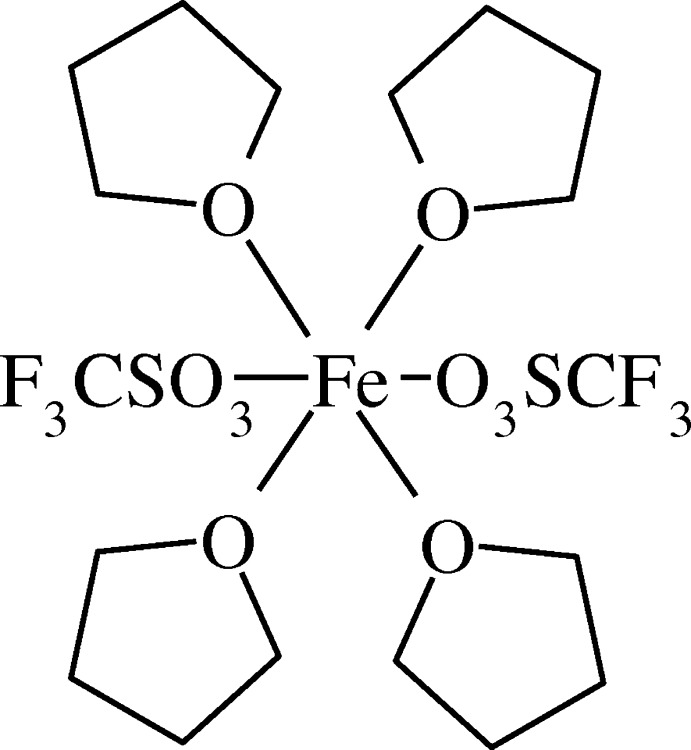


## Structural commentary   

**Figure 1:**
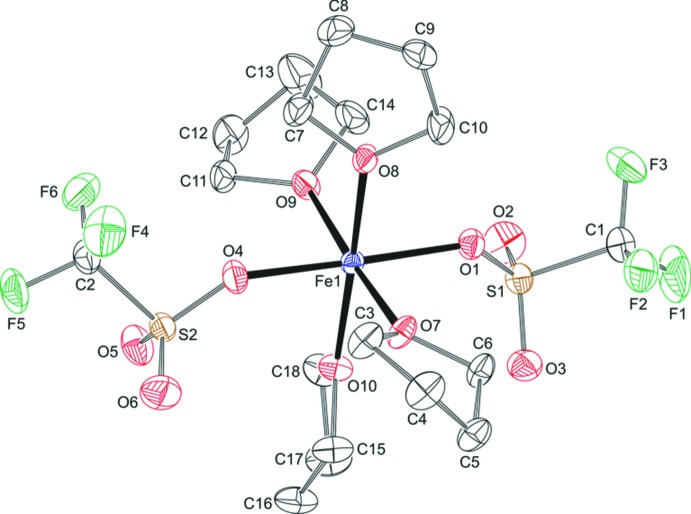
A view of the molecular structure of (I), with atom labelling. Displacement ellipsoids are drawn at the 50% probability level. For clarity, H atoms have been omitted.

**Figure 2:**
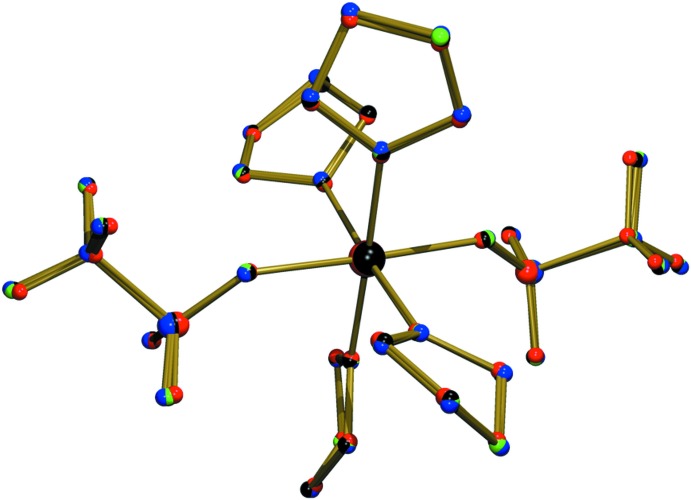
Overlay plot of the isostructural Co, Ni, and Zn complexes (Amel’chenkova *et al.*, 2006 ▸) with respect to the Fe complex (I). The coordinates of the Ni and Zn complexes have been inverted for this comparison. Hydrogen atoms are omitted for clarity. The quaternion fit algorithm (Mackay, 1984 ▸) as implemented in *PLATON* (Spek, 2009 ▸) was used for the preparation of the plot. Color scheme: Fe complex (blue),Co complex (green), Ni complex (red), and Zn complex (black).

A mol­ecular plot of (I) is shown in Fig. 1 ▸ with selected bond lengths and bond angles given in Table 1 ▸. The present Fe compound is isostructural to the corresponding Co, Ni and Zn compounds known from the literature (Amel’chenkova *et al.*, 2006 ▸). An isostructural Cu compound is mentioned in the same publication but no further details are given. An overlay of the isostructural compounds is presented in Fig. 2 ▸. The comparison of metal–oxygen distances in Table 2 ▸ follows the trend of effective ionic radii (Shannon, 1976 ▸) with 0.92 Å for octa­hedral Fe^2+^ (high-spin), 0.885 Å for Co^2+^ (high-spin), 0.83 Å for Ni^2+^ and 0.88 Å for Zn^2+^. From this comparison we can conclude that the Fe ion in (I) has a high-spin electronic configuration. It should also be noted that there are no significant differences in metal–oxygen distances between the partially negative triflate and the neutral THF.

In the octa­hedral compound (I), the triflate ligands are in *trans* positions and the equatorial plane is formed by O atoms of THF. The Fe atom is approximately in the equatorial plane at a distance of 0.0079 (3) Å from the least-squares plane of the THF oxygen atoms. The FeO_6_ octa­hedron is nearly undistorted with a quadratic elongation of 1.001 and an angle variance of 2.79°^2^ (Robinson *et al.*, 1971 ▸). To the best of our knowledge, the crystal structure of compound (I) is the first of a *trans* triflate Fe complex with an FeO_6_ chromophore. Similar complexes with N atoms in the equatorial plane are known from the literature. In the aceto­nitrile complex [Fe(CF_3_SO_3_)_2_(CH_3_CN)_4_], the core octa­hedron is similarly undistorted (Hagen, 2000 ▸), while the pyridine complex [Fe(CF_3_SO_3_)_2_(C_5_H_5_N)_4_] is slightly tetra­gonally compressed (Haynes *et al.*, 1986 ▸).

As expected, all four coordinated THF mol­ecules are puckered. The rings at O7 and O8 are best described as having an envelope conformation, the rings at O9 and O10 as being in a twist conformation. The O atoms are coordinated to the metal in a trigonal geometry with angle sums of 358.7 (2)–360.0 (2)°.

The two triflate ligands adopt a staggered conformation with O—S—C—F torsion angles between 56.6 (2) and 64.11 (19)°. The S—O distances to the coordinating oxygen atoms are significantly longer than to the non-coordinating oxygen atoms (Table 1 ▸). A search in the Cambridge Structural Database (update May 2019; Groom *et al.*, 2016 ▸) shows a large variation between 99.3 and 178.2° in S—O—metal bond angles for the weakly coordinating triflate ligand (1501 observations, non-disordered structures). The angles of 135.31 (9) and 142.51 (9)° in compound (I) are well within this range.

**Figure 3:**
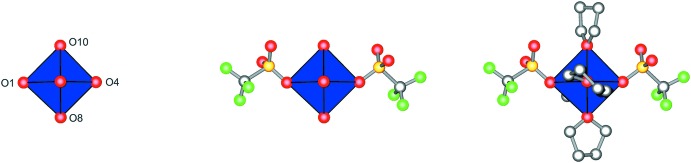
The approximate O_h_ symmetry of the FeO_6_ polyhedron (left, r.m.s.d. 0.0489 Å) is reduced by the tri­fluoro­methane­sulfonate coordination in the second coordination shell to approximate *C*_2_ (center, r.m.s.d. 0.1460 Å). If the coordinated THF mol­ecules are taken into consideration, the symmetry is only *C*_1_ (right). The algorithm of Pilati & Forni (1998 ▸) was used to calculate the r.m.s.d. values.

The octa­hedral symmetry of the inner-sphere coordination environment (see above) is reduced to approximate *C*_2_ symmetry by the arrangement of the triflate anion (Fig. 3 ▸). If the THF mol­ecules are considered as well, the overall symmetry reduces to *C*_1_. Despite the achiral ligands, the metal complex is thus chiral in the crystal.

## Supra­molecular features   

The crystal structure of (I) has a packing index (Kitajgorodskij, 1973 ▸) of only 68.7%, which is at the lower end of the 65–75% range expected for organic solids (Dunitz, 1995 ▸). Indeed, the packing is determined by only weak C—H⋯O inter­actions with the THF atoms as donors and the non-coordinated triflate oxygen atoms as acceptors (Table 3 ▸). Every mol­ecule of (I) is the donor and acceptor of three inter­molecular C—H⋯O hydrogen bonds and has thus a coordination number of six. This results in a three-dimensional network.

## Synthesis and crystallization   

The title compound was obtained from an experiment aimed at synthesizing an iron coordination compound based on an oxazine ligand. In a glovebox under a di­nitro­gen atmosphere, 4a,8a-di­methyl­octa­hydro-[1,4]oxazino[3,2-*b*][1,4]oxazine (159 mg, 0.923 mmol) and Fe(OTf)_2_·2MeCN (400 mg, 0.917 mmol) were placed in separate vials. The ligand was dissolved in THF (about 12 mL) and added to the vial containing Fe(OTf)_2_·2MeCN under gentle stirring. The color of the solution turned from black to dark red and stirring was maintained overnight at room temperature. The resulting compound was precipitated twice by dropwise addition of a concentrated THF solution into hexane. The slightly pink-colored supernatants were removed by deca­ntation. The precipitated solids were washed with hexa­nes and dried under vacuum. The deca­nted solutions were stored in a freezer at 238 K and over a month light-pink crystals slowly grew.

A second crystallization starting from the isolated precipitate in an 1:1 THF:hexane solution grew similar crystals over several months at 238 K. ^1^H-NMR in *d*_3_-MeCN showed no paramagnetic peaks but small diamagnetic peaks of THF (3.64, 1.79 ppm) and hexane (1.28, 0.89 ppm). ^19^F-NMR showed a single sharp peak at −79.36 ppm.

## Refinement   

Crystal data, data collection and structure refinement details are summarized in Table 4 ▸. H atoms were placed in calculated positions (C—H = 0.99 Å) and refined as riding with *U*_iso_(H) = 1.2*U*_eq_(C).

**Figure 4:**
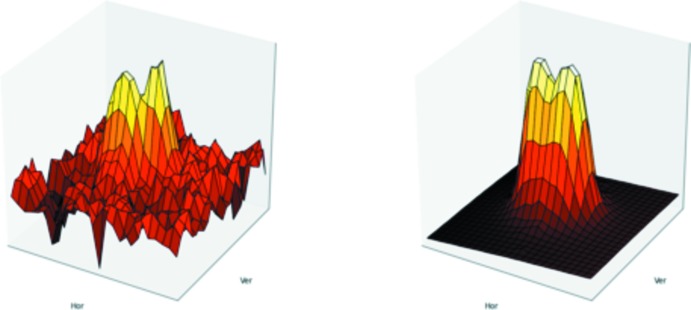
Height plot of the pixel intensities of reflection *hkl* = (5,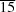,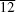). The central frame (scan width 0.3°) is shown. Observed intensities (left) and model intensities (right). A split-mosaic model was assumed for the prediction of the profile.

The reflection profiles in *Eval15* (Schreurs *et al.*, 2010 ▸) were based on a split-mosaic model. Two fragments were rotated by 0.56° with respect to each other. An example for a reflection profile is shown in Fig. 4 ▸.

Because (I) crystallizes in the Sohncke space group *P*2_1_2_1_2_1_ without second kind symmetry operations, it is susceptible for an absolute structure determination. A full-matrix refinement as inversion twin results in a Flack parameter of *x* = −0.001 (10) (Flack, 1983 ▸). Within standard uncertainties, the crystal structure can consequently be considered as enanti­omerically pure. The standard uncertainty is corrected for the different number of observations in the point group *versus* the Laue group symmetry (Sheldrick, 2015 ▸). If this correction is not applied (program *SHELXL97*, Sheldrick, 2008 ▸), the Flack parameter is *x* = −0.001 (8). Analysis of 2590 intensity quotients (Parsons *et al.* 2013 ▸) results in an absolute structure parameter of *z* = −0.001 (2). Similarly, a likelihood analysis on Bijvoet differences (Hooft *et al.*, 2008 ▸) gives an absolute structure parameter *y* = −0.000 (1). This analysis uses a *t*-value of 99, resulting in a slope of 0.885 and an inter­cept of −0.037. The student-*t* probability plot is linear with a correlation coefficient of 1.000. All of these different methods give a consistent result for the present crystal. The measurement of a second crystal results in *x* = 0.015 (11) from an inversion twin refinement, but very low standard uncertainties in the values of *z* = 0.015 (2) and *y* = 0.0012 (1) leave reasons for doubt concerning its enanti­opurity, although the Bijvoet difference related probabilities *P*2/*P*3 (true) are 1.000 and the probability *P*3 (false) is 0.000 in both crystals, suggesting that both crystals are enanti­opure.

## Supplementary Material

Crystal structure: contains datablock(s) I. DOI: 10.1107/S2056989019013094/vn2153sup1.cif

Structure factors: contains datablock(s) I. DOI: 10.1107/S2056989019013094/vn2153Isup2.hkl

CCDC reference: 1955192

Additional supporting information:  crystallographic information; 3D view; checkCIF report

## Figures and Tables

**Table 1:** Selected geometric parameters (Å, °)

S1—O2	1.4325 (16)	S2—O6	1.4290 (17)
S1—O3	1.4346 (15)	S2—O5	1.4329 (16)
S1—O1	1.4608 (14)	S2—O4	1.4608 (14)
			
O8—Fe1—O10	177.82 (6)	O9—Fe1—O7	175.98 (6)
O8—Fe1—O4	87.98 (5)	O8—Fe1—O1	89.70 (5)
O10—Fe1—O4	89.99 (6)	O10—Fe1—O1	92.31 (5)
O8—Fe1—O9	87.53 (6)	O4—Fe1—O1	177.54 (6)
O10—Fe1—O9	93.31 (5)	O9—Fe1—O1	89.86 (5)
O4—Fe1—O9	90.86 (6)	O7—Fe1—O1	88.90 (5)
O8—Fe1—O7	88.64 (6)	S1—O1—Fe1	135.31 (9)
O10—Fe1—O7	90.57 (5)	S2—O4—Fe1	142.51 (9)
O4—Fe1—O7	90.22 (6)		

**Table 2:** Comparison between the metal–oxygen distances of the Fe compound (I) and the isostructural Co, Ni and Zn compounds from the literature (Amel’chenkova *et al.*, 2006 ▸). The atom names of the Co and Ni complexes have been changed for consistency.

	*M* = Fe	*M* = Co	Δ Fe/Co	*M* = Ni	Δ Fe/Ni	*M* = Zn	Δ Fe/Zn
*M*—O1	2.1279 (14)	2.115 (3)	0.013 (3)	2.034 (3)	0.094 (3)	2.078 (3)	0.050 (3)
*M*—O4	2.1179 (14)	2.098 (3)	0.020 (3)	2.031 (3)	0.087 (3)	2.080 (3)	0.038 (3)
*M*—O7	2.1239 (12)	2.088 (3)	0.036 (3)	2.054 (2)	0.070 (2)	2.087 (3)	0.037 (3)
*M*—O8	2.1024 (13)	2.076 (3)	0.026 (3)	2.036 (2)	0.066 (2)	2.088 (3)	0.014 (3)
*M*—O9	2.1187 (13)	2.093 (3)	0.026 (3)	2.051 (2)	0.068 (2)	2.092 (3)	0.027 (3)
*M*—O10	2.1153 (13)	2.103 (3)	0.012 (3)	2.039 (3)	0.076 (3)	2.084 (3)	0.031 (3)

**Table 3:** Hydrogen-bond geometry (Å, °)

*D*—H⋯*A*	*D*—H	H⋯*A*	*D*⋯*A*	*D*—H⋯*A*
C3—H3*B*⋯O2^i^	0.99	2.55	3.514 (3)	164
C11—H11*B*⋯O5	0.99	2.59	3.412 (3)	140
C12—H12*A*⋯O3^ii^	0.99	2.49	3.388 (3)	151
C14—H14*A*⋯O2	0.99	2.51	3.429 (3)	155
C16—H16*B*⋯O6^iii^	0.99	2.56	3.476 (3)	154

Symmetry codes: (i) 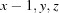; (ii) 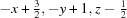; (iii) 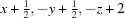.

**Table 4:** Experimental details

Crystal data	
Chemical formula	[Fe(CF_3_O_3_S)_2_(C_4_H_8_O)_4_]
*M*_r_	642.40
Crystal system, space group	Orthorhombic, *P*2_1_2_1_2_1_
Temperature (K)	150
*a*, *b*, *c* (Å)	8.6618 (3), 16.2610 (6), 19.0572 (4)
*V* (Å^3^)	2684.20 (14)
*Z*	4
Radiation type	Mo *K*α
μ (mm^−1^)	0.81
Crystal size (mm)	0.43 × 0.32 × 0.18

Data collection	
Diffractometer	Bruker Kappa APEXII CCD
Absorption correction	Multi-scan (*SADABS*; Krause *et al*., 2015 ▸)
*T*_min_, *T*_max_	0.652, 0.746
No. of measured, independent and observed [*I* > 2σ(*I*)] reflections	43720, 6166, 6027
*R*_int_	0.020
(sin θ/λ)_max_ (Å^−1^)	0.649

Refinement	
*R*[*F* ^2^ > 2σ(*F* ^2^)], *wR*(*F* ^2^), *S*	0.019, 0.051, 1.07
No. of reflections	6166
No. of parameters	335
H-atom treatment	H-atom parameters constrained
Δρ_max_, Δρ_min_ (e Å^−3^)	0.33, −0.28
Absolute structure	Refined as an inversion twin
Absolute structure parameter	−0.001 (10)

Computer programs: *APEX2* (Bruker, 2007 ▸), *PEAKREF* (Schreurs, 2016 ▸), *Eval15* (Schreurs *et al.*, 2010 ▸), initial coordinates from isostructural Zn complex (Amel’chenkova *et al.*, 2006 ▸), *SHELXL2018* (Sheldrick, 2015 ▸), *PLATON* (Spek, 2009 ▸) and *DRAWxtl* (Finger *et al.*, 2007 ▸).

## supplementary crystallographic information

### Crystal data

**Table:**

[Fe(CF_3_O_3_S)_2_(C_4_H_8_O)_4_]	*D*_x_ = 1.590 Mg m^−^^3^
*M**_r_* = 642.40	Mo *K*α radiation, λ = 0.71073 Å
Orthorhombic, *P*2_1_2_1_2_1_	Cell parameters from 40729 reflections
*a* = 8.6618 (3) Å	θ = 1.6–27.5°
*b* = 16.2610 (6) Å	µ = 0.81 mm^−^^1^
*c* = 19.0572 (4) Å	*T* = 150 K
*V* = 2684.20 (14) Å^3^	Block, light pink
*Z* = 4	0.43 × 0.32 × 0.18 mm
*F*(000) = 1328	

### Data collection

**Table:**

Bruker Kappa APEXII CCD diffractometer	6027 reflections with *I* > 2σ(*I*)
Radiation source: sealed tube	*R*_int_ = 0.020
φ and ω scans	θ_max_ = 27.5°, θ_min_ = 1.7°
Absorption correction: multi-scan (SADABS; Krause et al., 2015)	*h* = −11→11
*T*_min_ = 0.652, *T*_max_ = 0.746	*k* = −21→21
43720 measured reflections	*l* = −24→24
6166 independent reflections	

### Refinement

**Table:**

Refinement on *F*^2^	Secondary atom site location: difference Fourier map
Least-squares matrix: full	Hydrogen site location: difference Fourier map
*R*[*F*^2^ > 2σ(*F*^2^)] = 0.019	H-atom parameters constrained
*wR*(*F*^2^) = 0.051	*w* = 1/[σ^2^(*F*_o_^2^) + (0.0277*P*)^2^ + 0.6586*P*] where *P* = (*F*_o_^2^ + 2*F*_c_^2^)/3
*S* = 1.07	(Δ/σ)_max_ = 0.001
6166 reflections	Δρ_max_ = 0.33 e Å^−^^3^
335 parameters	Δρ_min_ = −0.28 e Å^−^^3^
0 restraints	Absolute structure: Refined as an inversion twin
Primary atom site location: structure-invariant direct methods	Absolute structure parameter: −0.001 (10)

### Special details

**Table:**

Geometry. All esds (except the esd in the dihedral angle between two l.s. planes) are estimated using the full covariance matrix. The cell esds are taken into account individually in the estimation of esds in distances, angles and torsion angles; correlations between esds in cell parameters are only used when they are defined by crystal symmetry. An approximate (isotropic) treatment of cell esds is used for estimating esds involving l.s. planes.
Refinement. Refined as a two-component inversion twin

### Fractional atomic coordinates and isotropic or equivalent isotropic displacement parameters (Å^2^)

**Table:**

	*x*	*y*	*z*	*U*_iso_*/*U*_eq_	
Fe1	0.50772 (3)	0.48861 (2)	0.86831 (2)	0.01643 (6)	
S1	0.81595 (5)	0.57825 (3)	0.93928 (2)	0.02277 (10)	
S2	0.32496 (6)	0.31474 (3)	0.81507 (2)	0.02265 (10)	
F1	0.9574 (2)	0.68741 (12)	1.01554 (11)	0.0680 (6)	
F2	0.7121 (2)	0.68477 (9)	1.03052 (7)	0.0470 (4)	
F3	0.8062 (3)	0.73954 (9)	0.93805 (9)	0.0590 (5)	
F4	0.06455 (18)	0.35905 (12)	0.75689 (10)	0.0558 (4)	
F5	0.14957 (18)	0.24184 (9)	0.72262 (9)	0.0480 (4)	
F6	0.2551 (2)	0.35272 (10)	0.68562 (7)	0.0508 (4)	
O1	0.65973 (16)	0.57880 (9)	0.91040 (7)	0.0240 (3)	
O2	0.93533 (19)	0.58245 (12)	0.88737 (9)	0.0406 (4)	
O3	0.83890 (17)	0.52029 (9)	0.99506 (8)	0.0324 (3)	
O4	0.34877 (17)	0.40267 (8)	0.82643 (8)	0.0274 (3)	
O5	0.45752 (17)	0.27184 (10)	0.78893 (9)	0.0353 (4)	
O6	0.2398 (2)	0.27486 (11)	0.86955 (9)	0.0428 (4)	
O7	0.38706 (15)	0.48776 (10)	0.96533 (6)	0.0238 (3)	
O8	0.35941 (17)	0.58263 (8)	0.83381 (7)	0.0256 (3)	
O9	0.62129 (17)	0.49769 (8)	0.77015 (7)	0.0264 (3)	
O10	0.64976 (15)	0.39087 (8)	0.90293 (7)	0.0235 (3)	
C1	0.8230 (3)	0.67820 (14)	0.98276 (12)	0.0350 (5)	
C2	0.1914 (3)	0.31710 (13)	0.74133 (11)	0.0296 (4)	
C3	0.2234 (2)	0.46794 (15)	0.97287 (10)	0.0305 (5)	
H3A	0.203548	0.410133	0.959313	0.037*	
H3B	0.159680	0.504403	0.943011	0.037*	
C4	0.1865 (2)	0.48144 (16)	1.05024 (11)	0.0354 (5)	
H4A	0.115020	0.438696	1.067968	0.042*	
H4B	0.139912	0.536288	1.058032	0.042*	
C5	0.3443 (2)	0.47496 (15)	1.08559 (10)	0.0308 (4)	
H5A	0.346138	0.504546	1.130987	0.037*	
H5B	0.374432	0.416917	1.093207	0.037*	
C6	0.4466 (2)	0.51605 (14)	1.03204 (9)	0.0272 (4)	
H6A	0.439699	0.576664	1.035736	0.033*	
H6B	0.555633	0.499216	1.038254	0.033*	
C7	0.2531 (3)	0.57493 (15)	0.77512 (11)	0.0349 (5)	
H7A	0.159543	0.544020	0.789144	0.042*	
H7B	0.302789	0.546152	0.735315	0.042*	
C8	0.2128 (3)	0.66148 (15)	0.75551 (12)	0.0358 (5)	
H8A	0.107456	0.664582	0.735575	0.043*	
H8B	0.287162	0.683703	0.720896	0.043*	
C9	0.2226 (3)	0.70770 (13)	0.82393 (12)	0.0318 (5)	
H9A	0.250509	0.765977	0.815906	0.038*	
H9B	0.123089	0.705398	0.849438	0.038*	
C10	0.3467 (3)	0.66399 (13)	0.86392 (13)	0.0417 (6)	
H10A	0.445941	0.693786	0.859551	0.050*	
H10B	0.319250	0.660390	0.914247	0.050*	
C11	0.6152 (3)	0.44132 (13)	0.71141 (10)	0.0274 (4)	
H11A	0.518112	0.448247	0.684649	0.033*	
H11B	0.622800	0.383601	0.727581	0.033*	
C12	0.7528 (3)	0.46439 (15)	0.66738 (12)	0.0393 (5)	
H12A	0.738428	0.447800	0.617830	0.047*	
H12B	0.848683	0.439136	0.685716	0.047*	
C13	0.7560 (4)	0.55698 (18)	0.67491 (16)	0.0593 (9)	
H13A	0.863015	0.577957	0.671068	0.071*	
H13B	0.692138	0.583316	0.638162	0.071*	
C14	0.6920 (4)	0.57395 (14)	0.74568 (11)	0.0407 (6)	
H14A	0.775102	0.591301	0.778098	0.049*	
H14B	0.613879	0.618318	0.743218	0.049*	
C15	0.6119 (3)	0.33496 (14)	0.95980 (11)	0.0316 (4)	
H15A	0.502945	0.317000	0.956510	0.038*	
H15B	0.628145	0.361845	1.005821	0.038*	
C16	0.7192 (3)	0.26300 (15)	0.95120 (13)	0.0376 (5)	
H16A	0.677552	0.222297	0.917507	0.045*	
H16B	0.739603	0.235545	0.996623	0.045*	
C17	0.8638 (3)	0.30493 (17)	0.92264 (14)	0.0417 (6)	
H17A	0.922310	0.332764	0.960436	0.050*	
H17B	0.932237	0.265169	0.898545	0.050*	
C18	0.7963 (2)	0.36611 (13)	0.87176 (12)	0.0292 (4)	
H18A	0.865616	0.414073	0.866134	0.035*	
H18B	0.779706	0.340471	0.825253	0.035*	

### Atomic displacement parameters (Å^2^)

**Table:**

	*U*^11^	*U*^22^	*U*^33^	*U*^12^	*U*^13^	*U*^23^
Fe1	0.01825 (11)	0.01659 (11)	0.01444 (10)	0.00166 (10)	0.00053 (9)	−0.00065 (8)
S1	0.0182 (2)	0.0244 (2)	0.0257 (2)	−0.00151 (17)	0.00011 (18)	−0.00155 (17)
S2	0.0229 (2)	0.0189 (2)	0.0261 (2)	−0.00106 (17)	−0.00181 (18)	−0.00144 (16)
F1	0.0548 (10)	0.0586 (11)	0.0907 (14)	−0.0162 (8)	−0.0327 (10)	−0.0222 (10)
F2	0.0648 (10)	0.0402 (8)	0.0360 (7)	0.0033 (7)	0.0047 (7)	−0.0135 (6)
F3	0.0976 (14)	0.0258 (7)	0.0536 (9)	−0.0106 (8)	−0.0009 (10)	0.0063 (7)
F4	0.0342 (7)	0.0635 (11)	0.0697 (11)	0.0195 (7)	−0.0173 (7)	−0.0151 (9)
F5	0.0478 (9)	0.0322 (7)	0.0640 (9)	−0.0105 (6)	−0.0218 (7)	−0.0118 (7)
F6	0.0648 (10)	0.0564 (9)	0.0311 (7)	−0.0160 (8)	−0.0116 (7)	0.0056 (7)
O1	0.0226 (6)	0.0237 (7)	0.0256 (6)	−0.0001 (5)	−0.0033 (5)	−0.0029 (5)
O2	0.0279 (7)	0.0495 (10)	0.0443 (10)	−0.0002 (7)	0.0124 (7)	−0.0004 (8)
O3	0.0293 (7)	0.0315 (7)	0.0364 (7)	0.0018 (6)	−0.0072 (6)	0.0046 (6)
O4	0.0284 (7)	0.0211 (7)	0.0326 (7)	0.0000 (5)	−0.0059 (6)	−0.0067 (6)
O5	0.0274 (7)	0.0271 (8)	0.0514 (9)	0.0054 (6)	−0.0033 (7)	−0.0092 (7)
O6	0.0478 (10)	0.0439 (9)	0.0368 (8)	−0.0087 (8)	0.0054 (8)	0.0099 (8)
O7	0.0173 (6)	0.0382 (8)	0.0160 (5)	−0.0010 (6)	0.0006 (4)	−0.0031 (6)
O8	0.0334 (8)	0.0201 (6)	0.0233 (6)	0.0088 (6)	−0.0091 (6)	−0.0057 (5)
O9	0.0397 (7)	0.0196 (7)	0.0199 (6)	−0.0031 (6)	0.0099 (5)	−0.0025 (5)
O10	0.0199 (7)	0.0233 (6)	0.0273 (6)	0.0039 (5)	0.0045 (5)	0.0053 (5)
C1	0.0411 (12)	0.0290 (10)	0.0351 (11)	−0.0086 (10)	−0.0066 (10)	−0.0027 (9)
C2	0.0285 (10)	0.0247 (9)	0.0356 (10)	−0.0027 (8)	−0.0070 (9)	−0.0049 (8)
C3	0.0171 (9)	0.0492 (13)	0.0253 (9)	−0.0037 (8)	0.0017 (7)	0.0003 (9)
C4	0.0244 (9)	0.0533 (14)	0.0284 (9)	0.0015 (10)	0.0073 (8)	0.0001 (10)
C5	0.0309 (10)	0.0433 (12)	0.0183 (8)	−0.0017 (9)	0.0034 (7)	0.0006 (8)
C6	0.0274 (9)	0.0374 (11)	0.0169 (8)	−0.0044 (8)	0.0002 (7)	−0.0043 (8)
C7	0.0416 (12)	0.0330 (11)	0.0301 (10)	0.0088 (10)	−0.0163 (9)	−0.0054 (9)
C8	0.0390 (12)	0.0357 (12)	0.0325 (11)	0.0092 (10)	−0.0090 (9)	0.0042 (9)
C9	0.0362 (11)	0.0213 (9)	0.0379 (11)	0.0063 (8)	−0.0023 (9)	0.0022 (8)
C10	0.0577 (15)	0.0241 (10)	0.0432 (12)	0.0170 (10)	−0.0213 (12)	−0.0150 (10)
C11	0.0372 (10)	0.0255 (10)	0.0195 (8)	−0.0029 (8)	0.0047 (8)	−0.0057 (7)
C12	0.0488 (14)	0.0415 (13)	0.0275 (10)	−0.0022 (10)	0.0154 (10)	−0.0072 (9)
C13	0.084 (2)	0.0427 (15)	0.0515 (16)	−0.0202 (15)	0.0368 (16)	−0.0027 (12)
C14	0.0679 (17)	0.0247 (10)	0.0295 (10)	−0.0127 (11)	0.0150 (11)	0.0011 (8)
C15	0.0301 (10)	0.0353 (11)	0.0294 (10)	0.0062 (9)	0.0039 (8)	0.0126 (8)
C16	0.0424 (12)	0.0317 (11)	0.0386 (11)	0.0109 (10)	0.0024 (10)	0.0136 (9)
C17	0.0284 (11)	0.0489 (14)	0.0479 (13)	0.0154 (10)	0.0012 (10)	0.0088 (11)
C18	0.0245 (9)	0.0269 (9)	0.0363 (10)	0.0057 (7)	0.0080 (9)	0.0020 (9)

### Geometric parameters (Å, º)

**Table:**

Fe1—O8	2.1024 (13)	C5—H5B	0.9900
Fe1—O10	2.1153 (13)	C6—H6A	0.9900
Fe1—O4	2.1179 (14)	C6—H6B	0.9900
Fe1—O9	2.1187 (13)	C7—C8	1.497 (3)
Fe1—O7	2.1239 (12)	C7—H7A	0.9900
Fe1—O1	2.1279 (14)	C7—H7B	0.9900
S1—O2	1.4325 (16)	C8—C9	1.507 (3)
S1—O3	1.4346 (15)	C8—H8A	0.9900
S1—O1	1.4608 (14)	C8—H8B	0.9900
S1—C1	1.825 (2)	C9—C10	1.497 (3)
S2—O6	1.4290 (17)	C9—H9A	0.9900
S2—O5	1.4329 (16)	C9—H9B	0.9900
S2—O4	1.4608 (14)	C10—H10A	0.9900
S2—C2	1.821 (2)	C10—H10B	0.9900
F1—C1	1.329 (3)	C11—C12	1.505 (3)
F2—C1	1.328 (3)	C11—H11A	0.9900
F3—C1	1.320 (3)	C11—H11B	0.9900
F4—C2	1.327 (3)	C12—C13	1.513 (4)
F5—C2	1.325 (2)	C12—H12A	0.9900
F6—C2	1.330 (3)	C12—H12B	0.9900
O7—C6	1.447 (2)	C13—C14	1.484 (3)
O7—C3	1.461 (2)	C13—H13A	0.9900
O8—C10	1.446 (2)	C13—H13B	0.9900
O8—C7	1.454 (2)	C14—H14A	0.9900
O9—C11	1.448 (2)	C14—H14B	0.9900
O9—C14	1.459 (3)	C15—C16	1.503 (3)
O10—C15	1.452 (2)	C15—H15A	0.9900
O10—C18	1.458 (2)	C15—H15B	0.9900
C3—C4	1.525 (3)	C16—C17	1.527 (3)
C3—H3A	0.9900	C16—H16A	0.9900
C3—H3B	0.9900	C16—H16B	0.9900
C4—C5	1.528 (3)	C17—C18	1.507 (3)
C4—H4A	0.9900	C17—H17A	0.9900
C4—H4B	0.9900	C17—H17B	0.9900
C5—C6	1.508 (3)	C18—H18A	0.9900
C5—H5A	0.9900	C18—H18B	0.9900
			
O8—Fe1—O10	177.82 (6)	C5—C6—H6B	110.9
O8—Fe1—O4	87.98 (5)	H6A—C6—H6B	109.0
O10—Fe1—O4	89.99 (6)	O8—C7—C8	104.97 (17)
O8—Fe1—O9	87.53 (6)	O8—C7—H7A	110.8
O10—Fe1—O9	93.31 (5)	C8—C7—H7A	110.8
O4—Fe1—O9	90.86 (6)	O8—C7—H7B	110.8
O8—Fe1—O7	88.64 (6)	C8—C7—H7B	110.8
O10—Fe1—O7	90.57 (5)	H7A—C7—H7B	108.8
O4—Fe1—O7	90.22 (6)	C7—C8—C9	103.87 (17)
O9—Fe1—O7	175.98 (6)	C7—C8—H8A	111.0
O8—Fe1—O1	89.70 (5)	C9—C8—H8A	111.0
O10—Fe1—O1	92.31 (5)	C7—C8—H8B	111.0
O4—Fe1—O1	177.54 (6)	C9—C8—H8B	111.0
O9—Fe1—O1	89.86 (5)	H8A—C8—H8B	109.0
O7—Fe1—O1	88.90 (5)	C10—C9—C8	104.11 (18)
O2—S1—O3	116.30 (10)	C10—C9—H9A	110.9
O2—S1—O1	114.09 (9)	C8—C9—H9A	110.9
O3—S1—O1	114.30 (9)	C10—C9—H9B	110.9
O2—S1—C1	104.29 (11)	C8—C9—H9B	110.9
O3—S1—C1	104.11 (10)	H9A—C9—H9B	109.0
O1—S1—C1	101.34 (10)	O8—C10—C9	106.67 (17)
O6—S2—O5	116.43 (11)	O8—C10—H10A	110.4
O6—S2—O4	114.17 (10)	C9—C10—H10A	110.4
O5—S2—O4	114.51 (10)	O8—C10—H10B	110.4
O6—S2—C2	104.02 (11)	C9—C10—H10B	110.4
O5—S2—C2	104.55 (10)	H10A—C10—H10B	108.6
O4—S2—C2	100.57 (9)	O9—C11—C12	104.14 (17)
S1—O1—Fe1	135.31 (9)	O9—C11—H11A	110.9
S2—O4—Fe1	142.51 (9)	C12—C11—H11A	110.9
C6—O7—C3	109.25 (14)	O9—C11—H11B	110.9
C6—O7—Fe1	125.96 (11)	C12—C11—H11B	110.9
C3—O7—Fe1	124.38 (11)	H11A—C11—H11B	108.9
C10—O8—C7	109.61 (15)	C11—C12—C13	102.1 (2)
C10—O8—Fe1	125.99 (12)	C11—C12—H12A	111.3
C7—O8—Fe1	124.39 (12)	C13—C12—H12A	111.3
C11—O9—C14	107.83 (14)	C11—C12—H12B	111.3
C11—O9—Fe1	128.44 (12)	C13—C12—H12B	111.3
C14—O9—Fe1	122.42 (12)	H12A—C12—H12B	109.2
C15—O10—C18	109.12 (15)	C14—C13—C12	105.3 (2)
C15—O10—Fe1	124.88 (12)	C14—C13—H13A	110.7
C18—O10—Fe1	125.93 (11)	C12—C13—H13A	110.7
F3—C1—F2	107.6 (2)	C14—C13—H13B	110.7
F3—C1—F1	108.4 (2)	C12—C13—H13B	110.7
F2—C1—F1	107.59 (19)	H13A—C13—H13B	108.8
F3—C1—S1	112.09 (16)	O9—C14—C13	106.82 (18)
F2—C1—S1	111.02 (15)	O9—C14—H14A	110.4
F1—C1—S1	110.05 (18)	C13—C14—H14A	110.4
F5—C2—F4	108.00 (19)	O9—C14—H14B	110.4
F5—C2—F6	107.51 (18)	C13—C14—H14B	110.4
F4—C2—F6	107.37 (19)	H14A—C14—H14B	108.6
F5—C2—S2	111.21 (15)	O10—C15—C16	105.46 (17)
F4—C2—S2	111.38 (15)	O10—C15—H15A	110.7
F6—C2—S2	111.18 (15)	C16—C15—H15A	110.7
O7—C3—C4	105.50 (16)	O10—C15—H15B	110.7
O7—C3—H3A	110.6	C16—C15—H15B	110.7
C4—C3—H3A	110.6	H15A—C15—H15B	108.8
O7—C3—H3B	110.6	C15—C16—C17	101.44 (19)
C4—C3—H3B	110.6	C15—C16—H16A	111.5
H3A—C3—H3B	108.8	C17—C16—H16A	111.5
C3—C4—C5	103.22 (16)	C15—C16—H16B	111.5
C3—C4—H4A	111.1	C17—C16—H16B	111.5
C5—C4—H4A	111.1	H16A—C16—H16B	109.3
C3—C4—H4B	111.1	C18—C17—C16	101.86 (17)
C5—C4—H4B	111.1	C18—C17—H17A	111.4
H4A—C4—H4B	109.1	C16—C17—H17A	111.4
C6—C5—C4	101.37 (16)	C18—C17—H17B	111.4
C6—C5—H5A	111.5	C16—C17—H17B	111.4
C4—C5—H5A	111.5	H17A—C17—H17B	109.3
C6—C5—H5B	111.5	O10—C18—C17	104.95 (17)
C4—C5—H5B	111.5	O10—C18—H18A	110.8
H5A—C5—H5B	109.3	C17—C18—H18A	110.8
O7—C6—C5	104.12 (16)	O10—C18—H18B	110.8
O7—C6—H6A	110.9	C17—C18—H18B	110.8
C5—C6—H6A	110.9	H18A—C18—H18B	108.8
O7—C6—H6B	110.9		
			
O2—S1—O1—Fe1	83.66 (14)	O7—C3—C4—C5	−21.8 (2)
O3—S1—O1—Fe1	−53.60 (15)	C3—C4—C5—C6	37.3 (2)
C1—S1—O1—Fe1	−164.91 (12)	C3—O7—C6—C5	27.1 (2)
O6—S2—O4—Fe1	93.74 (17)	Fe1—O7—C6—C5	−160.09 (13)
O5—S2—O4—Fe1	−44.07 (19)	C4—C5—C6—O7	−39.6 (2)
C2—S2—O4—Fe1	−155.53 (15)	C10—O8—C7—C8	18.0 (3)
O2—S1—C1—F3	56.6 (2)	Fe1—O8—C7—C8	−161.93 (14)
O3—S1—C1—F3	178.93 (19)	O8—C7—C8—C9	−30.8 (2)
O1—S1—C1—F3	−62.2 (2)	C7—C8—C9—C10	32.0 (3)
O2—S1—C1—F2	176.88 (16)	C7—O8—C10—C9	2.3 (3)
O3—S1—C1—F2	−60.75 (18)	Fe1—O8—C10—C9	−177.78 (14)
O1—S1—C1—F2	58.15 (17)	C8—C9—C10—O8	−21.4 (3)
O2—S1—C1—F1	−64.11 (19)	C14—O9—C11—C12	30.3 (2)
O3—S1—C1—F1	58.26 (19)	Fe1—O9—C11—C12	−162.73 (15)
O1—S1—C1—F1	177.16 (17)	O9—C11—C12—C13	−37.2 (3)
O6—S2—C2—F5	−60.00 (19)	C11—C12—C13—C14	30.6 (3)
O5—S2—C2—F5	62.60 (18)	C11—O9—C14—C13	−10.8 (3)
O4—S2—C2—F5	−178.42 (16)	Fe1—O9—C14—C13	−178.7 (2)
O6—S2—C2—F4	60.52 (19)	C12—C13—C14—O9	−13.0 (4)
O5—S2—C2—F4	−176.87 (16)	C18—O10—C15—C16	−13.7 (2)
O4—S2—C2—F4	−57.89 (18)	Fe1—O10—C15—C16	163.46 (14)
O6—S2—C2—F6	−179.78 (16)	O10—C15—C16—C17	33.9 (2)
O5—S2—C2—F6	−57.17 (17)	C15—C16—C17—C18	−41.0 (2)
O4—S2—C2—F6	61.81 (17)	C15—O10—C18—C17	−12.8 (2)
C6—O7—C3—C4	−3.0 (2)	Fe1—O10—C18—C17	170.13 (15)
Fe1—O7—C3—C4	−175.97 (14)	C16—C17—C18—O10	33.4 (2)

### Hydrogen-bond geometry (Å, º)

**Table:**

*D*—H···*A*	*D*—H	H···*A*	*D*···*A*	*D*—H···*A*
C3—H3*B*···O2^i^	0.99	2.55	3.514 (3)	164
C11—H11*B*···O5	0.99	2.59	3.412 (3)	140
C12—H12*A*···O3^ii^	0.99	2.49	3.388 (3)	151
C14—H14*A*···O2	0.99	2.51	3.429 (3)	155
C16—H16*B*···O6^iii^	0.99	2.56	3.476 (3)	154

Symmetry codes: (i) *x*−1, *y*, *z*; (ii) −*x*+3/2, −*y*+1, *z*−1/2; (iii) *x*+1/2, −*y*+1/2, −*z*+2.
